# Surface-Enhanced Raman Spectroscopy at the Interface between Drug Discovery and Personalized Medicine

**DOI:** 10.1021/acs.jpcc.4c04006

**Published:** 2024-09-24

**Authors:** Lamyaa M. Almehmadi, Igor K. Lednev

**Affiliations:** † Department of Materials Science and Engineering, Massachusetts Institute of Technology, Cambridge, Massachusetts 02139, United States; ‡ Department of Chemistry, University at Albany, State University of New York, 1400 Washington Avenue, Albany, New York 12222, United States

## Abstract

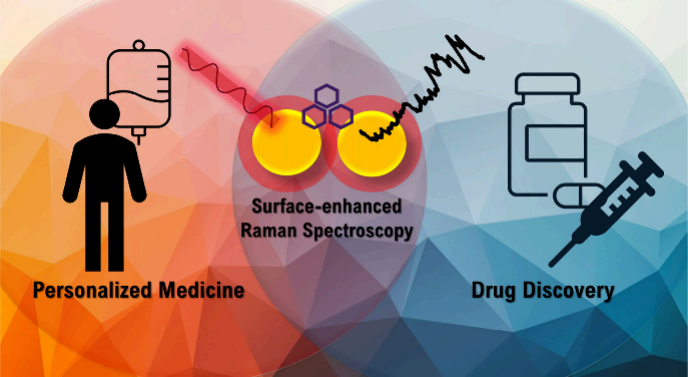

Personalized medicine and drug discovery are different, yet overlapping, fields, and information from each field is exchanged to improve the other. The current methods used for devising personalized therapeutic plans and developing drug discovery applications are costly, time-consuming, and complex; thus, their applicability is limited in both fields. However, surface-enhanced Raman spectroscopy (SERS) offers potential solutions to current challenges. This Mini-Review explores the utility of SERS in drug discovery and personalized medicine. The Mini-Review starts with a brief overview of these fields, including the main challenges and current methods, and then explores examples where SERS has been used to overcome some of the main challenges in both fields. It ends with brief conclusions, perspectives, and current challenges limiting the practical application of SERS.

## 1 Introduction

Personalized medicine and drug discovery substantially rely on technologies that facilitate the rapid and effective identification and analysis of molecules and molecular systems. From detecting validated biomarkers to monitoring treatment responses, drug discovery and personalized medicine synergistically interact to enable optimal therapeutics and well-informed individualized therapeutic plans for patients. However, the high cost, time requirements, and complexity of current methods can limit their applicability in these fields.^1,2^ Therefore, new technologies must be actively sought to improve the manner in which personalized medicine and drug discovery are conducted. Because of its label-free and rapid analysis, ultrahigh sensitivity, and requirement for only traces of liquid samples, surface-enhanced Raman spectroscopy (SERS) has emerged as a promising technique for application to personalized medicine and drug discovery. A recent comprehensive review article illustrated the great potential of SERS for personalized medicine.^3^ Several other research articles showcased the potential of SERS to be used for drug discovery applications.^1,4^

In the past 50 years, SERS has been a transformative technique that enables the label-free detection of single molecules^5,6^ by intensifying Raman signals via electromagnetic (EM) and chemical enhancement (CE) mechanisms.^7^ The CE mechanism accounts for approximately 10^2^ of the overall Raman signal enhancement.^3^ In the CE mechanism, when a molecule adsorbs on a SERS substrate surface, charge is transferred, changing the polarizability and thereby amplifying the Raman signal.^8^ However, the EM mechanism-derived Raman signal enhancement can reach 10^11^.^3^ This substantial enhancement relies on the localized surface plasmon resonance (LSPR) field generated when the incident light’s wavelength exceeds the particle size, causing plasmons to oscillate locally at a frequency termed as the LSPR.^9^ This plasmonic field arises from the oscillation of “free conduction electrons” on a metal surface because of their interaction with electromagnetic waves.^8^ Notably, this enhancement is limited to molecules in proximity to the surface.^10^ Therefore, to be detected, molecules of interest must be near the surface.^10^ The ultrasensitivity of SERS, which can be used to detect individual molecules, is attributed to molecules residing at hotspots,^11^ which are localized regions where the plasmonic field is strongly enhanced between metallic junctions.^11^ Substantially enhanced Raman signals while preserving rich molecular vibrations (fingerprints) have sparked interest in leveraging SERS for practical applications in various fields, such as medical diagnostics, forensics, and pharmaceuticals.^12−14^

**Figure 1:**
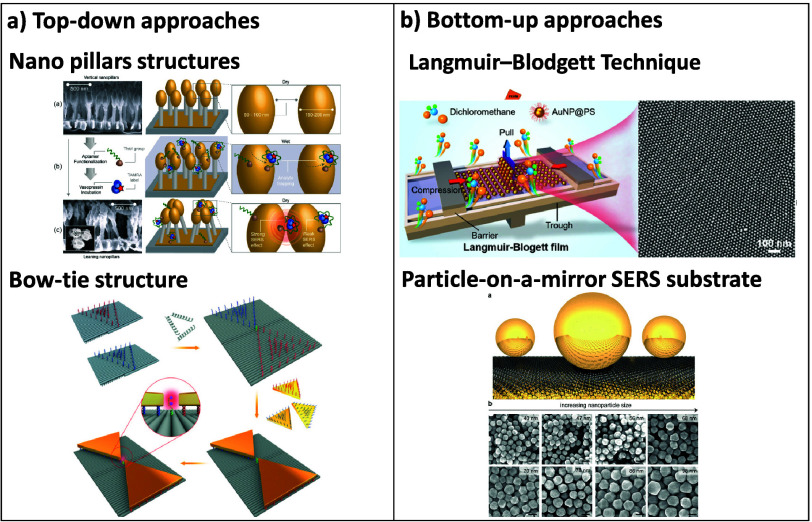
Plasmonic SERS substrates manufactured via top-down and bottom-up fabrication approaches. (a) Top-down approaches enable the fabrication of highly resolved nanostructures, such as nanopillars (reproduced from ref (16). Copyright 2013, American Chemical Society) and bowties (reproduced from ref (17). Copyright 2018, Wiley-VCH Verlag GmbH & Co. KGaA, Weinheim). (b) Bottom-up approaches, such as the Langmuir–Blodgett technique (reproduced from ref (29). Copyright 2022, Elsevier Ltd.) and the nanoparticle-on-a-mirror approach (licensed under CC-BY. Reproduced from ref (23)), are more accessible and facilitate the controlled aggregation of nanoparticles that are simpler to fabricate.

The fabrication of SERS substrates is crucial for enhancing Raman signals, and the fabrication of tailored SERS substrates, typically comprising nanostructured metals, such as gold or silver, has facilitated the detection of extremely low molecular concentrations in various environments.^2^ SERS substrates can be manufactured via either top-down or bottom-up approaches (Figure 1).^15^ Top-down methods, such as electron beam lithography, yield highly resolved nanostructures possessing good manufacturing reproducibility.^15^ Examples of SERS substrates manufactured using top-down methods include nanopillars that form hotspots when liquid samples are evaporated from the substrate and bowtie structures possessing predetermined gap widths (Figure 1a).^16,17^ In contrast, bottom-up methods involve colloidal dispersions, which are more accessible and can be easily produced in laboratories; however, such methods are often difficult to reproduce because of the random aggregation of nanoparticles.^18^ Nonetheless, a few examples of reproducible colloidal dispersion-based SERS substrates have been reported.^19^ Other examples of SERS substrates manufactured using bottom-up methods include those made with the Langmuir–Blodgett technique through the self-assembly of a monolayer of nanoparticles and the nanoparticle-on-a-mirror approach (Figure 1b).^20−23^ Recent developments in SERS substrate manufacturing have also enabled point-of-care applications using paper-based substrates and other wearable materials, allowing for continuous monitoring and *in situ* testing.^24,25^ In addition, lab-on-a-chip platforms based on SERS were also developed by Popp and coworkers, allowing high-throughput measurements under reproducible conditions.^26−28^

Exciting research developments showcasing SERS for personalized medicine and drug discovery applications have been reported, along with comprehensive review articles focused on the use of SERS for personalized medicine.^2,30,31^ Despite existing reviews on SERS for personalized medicine, none cover its dual role in both fields. This Mini-Review addresses this gap, showcasing SERS’s versatility in meeting technological needs for drug discovery and personalized medicine.

## 2 Drug Discovery and Personalized Medicine Fields

Drug discovery is a sophisticated and complex scientific undertaking that aims to identify novel and efficacious therapeutic agents for treating diseases and health conditions.^32^ Drug discovery encompasses a multistep process, starting from identifying promising therapeutic targets to developing and evaluating potential drug candidates.^33^ Drug discovery is characterized by its lengthy duration and substantial cost, approximately 12–15 years and $1 billion, respectively, for a single drug to reach the market.^33^ Furthermore, the high failure rate of drug candidates amplifies the timeline and cost associated with the process.^33^ Consequently, innovative methods and approaches must be devised to streamline drug discovery by reducing both the timeline and the expenses while ensuring the highly reliable identification of safe and effective drugs. Spectroscopic and spectrometric methods, such as nuclear magnetic resonance (NMR) and fluorescence spectroscopies and mass spectrometry, respectively, are established drug discovery techniques.^33^ Notably, because of its potentially superior performance to overcome some limitations encountered using current standard methods, Raman spectroscopy, specifically SERS, has gained traction in drug discovery literature.^1,14^

Personalized medicine focuses on providing treatment tailored to the needs of each patient rather than solely relying on classifying the illness.^34^ One main aspect is that personalized medicine focuses on detecting and monitoring validated biomarkers to precisely diagnose and classify health conditions. Another important aspect is that personalized medicine involves monitoring responses to therapeutics in individual patients. Such monitoring focuses on providing effective treatment while minimizing the risk of drug toxicity.^30^ Currently, clinical tests targeting precise disease diagnoses are time-consuming and expensive, limiting rapid responses to life-threatening illnesses and risking inaccurate diagnoses. In addition, therapeutic drug monitoring is currently performed using complex analytical techniques assigned to central laboratories, such as immunoassays or liquid/gas chromatography coupled with mass spectrometry.^35^ These techniques are challenging, as they are costly and time-consuming, require skilled personnel, and present limited accessibility or point-of-care operation.^35^ In addition, these methods typically require substantial sample volumes, presenting risks to patient health and limiting practicality.^35^ SERS is an alternative technique that enables the continuous tracking of validated biomarkers and therapeutic responses, thus overcoming the current challenges.

## 3 SERS Applications for Drug Discovery and Personalized Medicine

In this section, major developments where SERS is a promising technology for drug discovery and personalized medicine are explored. Although several examples are available in the literature, this Mini-Review sheds light on SERS applicability to both fields, specifically in an interchangeable manner. We focus on SERS applications for detecting and monitoring validated biomarkers, metabolites, and drug molecules in biological matrices, particularly in bodily fluids and cells. Examples of point-of-care and wearable SERS sensors are also discussed.

### 3.1 Detection and Monitoring of Validated Biomarkers and Metabolites

Validated biomarkers offer a path toward understanding disease progression, precise diagnosis, and, consequently, disease classification.^2,37^ In addition, validated biomarkers could be therapeutic targets.^1^ Therefore, methods enabling the detection and monitoring of validated biomarkers could be used for personalized medicine and drug discovery applications. Furthermore, recent studies on the global human metabolic network have demonstrated that imbalances in metabolites are associated with corresponding changes in gene expression.^31^ This, in turn, leads to modifications in protein-signaling pathways, where identified proteins function as biomarkers for phenotyping diseases.^31^ Other disease-related biomarkers include various biomolecules, such as DNA/RNA and cells.^37^ Technologies enabling the high-throughput generation of extensive biological data sets (omics), including the genome, proteome, metabolome, epigenome, transcriptome, and exposome, among other factors, are essential for advancing personalized medicine and accommodating individual variations.^31^ Cutshaw et al. extensively reviewed the use of SERS as an omics-based method for metabolic profiling in precision medicine.^31^ The review explored the use of SERS as a label and label-free method for detecting metabolic changes. Specific examples of the use of SERS for detecting and monitoring biomarkers and metabolites in personalized medicine and drug discovery are further discussed in this subsection.

The continuous monitoring of biomarker changes to evaluate how patients respond to treatment or develop resistance is crucial for individualized patient care. Current methods targeting bacterial infection diagnoses are time-consuming, requiring several hours of multistep procedures.^38^ Similarly, the evaluation of antibiotic susceptibility is challenging, requiring time-consuming tests for up to 8 h.^38^ However, SERS-based tests can determine antibiotic susceptibility in only 1–2 h.^38^ Furthermore, SERS can be used to phenotype bacteria and identify specific byproducts linked to cellular stress and the viability of isolated organisms.^38^ The latter can be probed following a label-free approach targeting changes in SERS spectra, indicating biomolecular changes due to the altered composition of bacteria’s outer membrane biomolecules, such as proteins, lipids, and fatty acids.^38^ Dina et al. comprehensively reviewed the use of SERS for antibiotic susceptibility testing.^38^ These examples demonstrate the potential of SERS to address the challenges associated with current methods. The adoption of SERS in clinical settings could significantly enhance personalized care and improve healthcare responses.

The use of SERS for monitoring metabolites is an effective approach for phenotyping diseases. Zhang et al. used SERS for diagnosing and classifying amyotrophic lateral sclerosis (ALS) according to metabolic changes in plasma samples.^39^ DNA/RNA-associated SERS bands were more intense in ALS patients compared to healthy donors and were correlated with ALS-associated genetic mutations.^39^ In addition, other spectral changes related to altered amino-acid metabolic pathways were detected.^39^ The same group also used SERS, specifically DNA/RNA Raman signals, in ALS prognosis to identify factors related to the relatively short survival of ALS patients.^40^ The authors also found that the intensity ratio of Raman bands of glycogen- and lactose-to-d-mannose in the short survival group was different, suggesting abnormal glucose metabolism in ALS progression, which is also supported by the ALS disease literature.^40^ The use of SERS for monitoring metabolic changes could provide deeper insight into diseases by monitoring the overall changes in the biochemical composition of a clinically relevant biofluid.

Similarly, Duan et al. combined SERS with NMR spectroscopy to reveal markers for acute myeloid leukemia (AML), which is characterized by sophisticated cytogenetic and molecular abnormalities in myeloid progenitor cells.^41^ These abnormalities are used for predicting and stratifying risk in AML prognoses.^41^ Clinically, bone marrow collected from AML patients is genetically and cytogenetically analyzed.^41^ SERS spectral variations due to changes in biochemical molecules, such as glucose, lipids, amino acids, and nucleic acids, enabled patients’ serum samples to be analyzed using SERS for classifying AML into its different subtypes.^41^ The authors predicted that SERS could be used for monitoring disease progression and conducting prognostic assessments.^41^ SERS is shown to potentially be a novel analytical method for detecting and monitoring biomarkers and metabolites for personalized medicine applications. Nevertheless, it is important to mention that analytical approaches for personalized medicine are still not fully developed and require comprehensive studies to determine the accuracy and relevance of biomarkers, treatments, and other factors affecting analytical health assessment at the individual patient level. In a recent perspective, Krylov and coauthors discussed the challenges in the current analytical approaches, specifically for predicting chemoresistance based on established biomarkers at the individual patient level.^36^

Disease-related biomarkers can be used as therapeutic targets. Almehmadi et al. used SERS to identify hits, an important drug discovery step, for detecting the binding between potential drug molecules (peptides) and a therapeutic RNA target correlated with myotonic dystrophy type 2 using a label-free approach.^1^ In the process of hit identification, potential drug molecules are selected based on their ability to bind to a target molecule. Current methods used for this step include fluorescence spectroscopy, surface plasmon resonance spectroscopy, and ELISA. These methods are time-consuming and resource-intensive and often require sample labeling. SERS, however, offers the opportunity to screen drug molecules using small amounts of sample and using a label-free approach.

**Figure 2:**
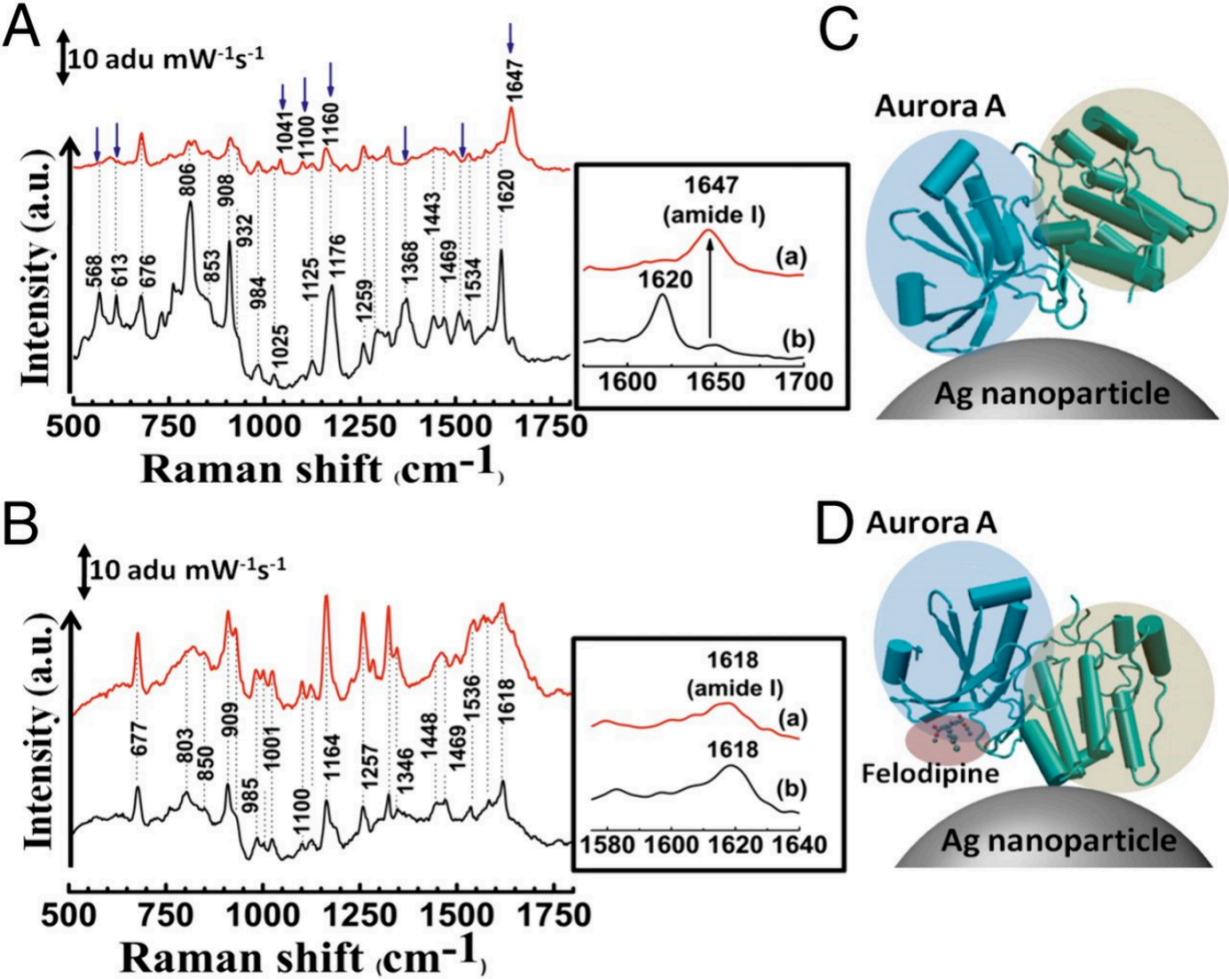
Detection of the binding site of the antihypertensive medication, felodipine, to its target protein, oncogenic Aurora A kinase, using label-free SERS by monitoring changes in the protein’s spectral bands. Reproduced with permission from ref (4).

Karthigeyan et al. effectively combined SERS with molecular docking calculations to pinpoint the binding sites of small drug molecules on important proteins for therapeutic applications (Figure 2).^4^ The authors highlighted SERS’s capability by examining the interaction between an antihypertensive medication, felodipine, and the oncogenic Aurora A kinase protein.^4^ In addition, Schultz et al. used SERS and tip-enhanced Raman spectroscopy (TERS) for detecting the specific binding between a ligand and a membrane protein receptor for reducing off-target effects that could complicate drug discovery applications.^42^ The authors correlated the spectral variation obtained using SERS and TERS as a method for evaluating ligand specificity.^42^ The ability to pinpoint binding sites and detect nonspecific binding highlights SERS’s precision in identifying key interactions between drug molecules and therapeutic targets. Thus, the use of SERS could potentially improve the drug screening process during the early drug discovery steps.

In addition, the detection of changes in cells’ biochemical compositions offers a means for personalized diagnoses. For example, Campbell et al. used a SERS microsensor to probe the cellular pH of organoids.^43^ Reportedly, changes in cellular pH indicate biological changes related to metabolic activities and immune responses.^43^ Their study is particularly promising, as SERS analysis of human explant organoids grown from patient samples offers the opportunity for the extended monitoring of therapeutic effects.^2^

### 3.2 Detection and Monitoring of Drug Molecules in Biological Matrices

Monitoring changes of drug concentrations in biological matrices, including bodily fluids and cells, is crucial for understanding drug mechanisms and evaluating the dosages required for optimal effectiveness while ensuring a safe treatment plan.^30^ Monitoring is particularly important for drugs possessing a narrow therapeutic index and when overdosing can be fatal.^30^ Moreover, during clinical trials in drug discovery, the monitoring of drug doses and their effects is essential.^44^ Similarly, the investigation of the effects of therapeutic drugs at the cellular level offers means for understanding drug mechanisms helpful for drug discovery.^45^

In practice, therapeutic monitoring determines the drug concentration in biological matrices (e.g., serum), as changes in drug concentration are related to drug activities.^46^ In some cases, the correlation of drug doses with serum concentrations is difficult to determine and could exhibit significant patient-to-patient variability.^47^ In addition, there are several therapeutics, such as antiepileptics, immunosuppressants, antibiotics, and cytotoxic drugs, that require patients to undergo continuous drug monitoring.^30^ Therefore, rapid, inexpensive, portable, multiplex, near-real-time, and sensitive technology is required for determining drug concentrations in biological samples, and SERS has been shown to satisfy these requirements. In this subsection, examples of the application of SERS for detecting and monitoring therapeutic drugs in bodily fluids and cells are discussed.

Studies investigating the application of SERS in detecting therapeutic drugs across various types of bodily fluids and for different health conditions underscore the remarkable versatility and potential of this technique. Panikar et al. used graphene oxide-based SERS substrates to detect widely used chemotherapeutic drugs, paclitaxel and cyclophosphamide,^48^ and achieved detection limits of 0.15 and 5 nM, respectively, in blood serum samples.^48^ One of the main challenges when using SERS for analyzing complex biological matrices is the nonspecific binding of biomolecules to the SERS substrate. Nonspecific binding can prevent small drug molecules from accessing the substrate surface, which significantly limits their detection, reducing the sensitivity and specificity of the analysis. To overcome this issue, the authors reduced surface fouling by incorporating a zwitterionic molecule, l-cysteine, which functions as a brush to reduce nonspecific interactions of the serum protein with the SERS substrate surface.^48^

In another study, Meneghetti et al. used a SERS-based sensor for detecting an anticancer drug in plasma samples spiked with different concentrations of erlotinib, a chemotherapeutic known for its high interpatient variability responses,^49^ and revealed that submilliliter of samples was sufficient for the detection of the drug in the nanomolar range.^49^ In this study, instead of using l-cysteine, the authors used the click chemistry reaction to enable competitive binding of the drug molecule to plasmonic nanostructures.^49^ Specifically, the authors utilized the carbon triple bond in the erlotinib structure for the click chemistry reaction for direct binding between the drug molecule and the plasmonic surface.^49^ Both studies highlight the effectiveness of SERS for detecting small drug molecules in biological matrices by either employing a surface modification step or leveraging the intrinsic properties of the drug molecules to mitigate the complexities introduced by other biomolecules in body fluids. While these approaches offer potential solutions to enable drug molecule detection in body fluids, the applicability of the developed SERS platforms for drug detection in all types of body fluids has not yet been demonstrated. Also, the utilization of intrinsic properties of drug molecule structures, such as for click chemistry, offers a selective advantage; however, it cannot be generalized to all types of drug molecules.

The combination of SERS with separation techniques and adaptation of SERS substrates with selective recognition components are alternative approaches, offering potential remedies for overcoming challenges arising from interference in biofluids. To overcome the challenges of saliva interferents, such as thiocyanate, Farquharson et al. used silver-doped sol–gel-filled capillaries, which generate SERS scattering and provide a means for separating chemicals,^50^ and achieved a detection limit of 2 μg/mL for a chemotherapeutic drug, fluorouracil, which was detected in under 5 min.

The use of the multivariate analysis method to overcome the complexity of the SERS signal is another alternative option. Subaihi et al. used statistical analysis to detect and quantify propranolol concentrations spiked in human serum samples without the need for sample pretreatment.^51^ The use of statistical analysis offers a simplified method for analyzing complex patterns in the SERS spectra. While this approach presents a promising solution, the variability associated with SERS limits the applicability of this approach. Jaworska et al. critically reviewed the potential and current challenges of SERS for therapeutic drug monitoring.^30^

**Figure 3:**
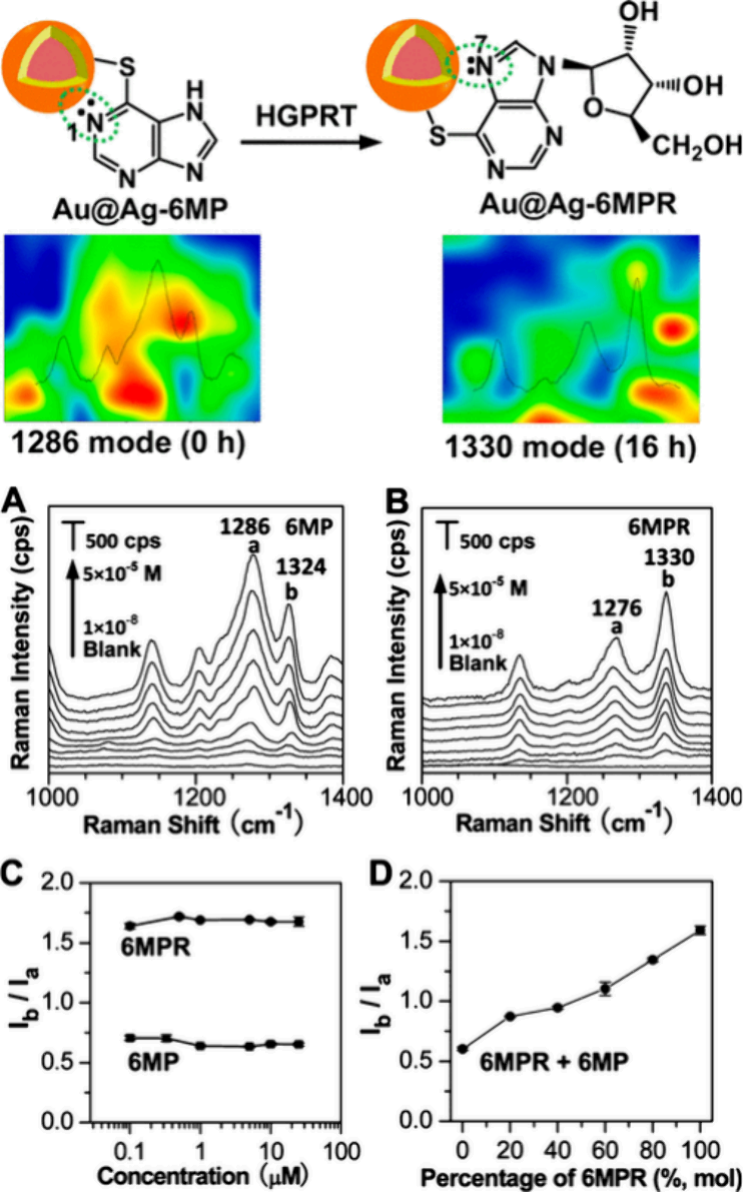
Application of label-free SERS for monitoring the cellular metabolism of a cancer therapeutic by detecting spectral changes associated with changes in the absorption of the drug molecule on nanoparticle surfaces. Reproduced from ref (54). Copyright 2014, American Chemical Society.

In the case of monitoring drug molecules at the cellular level, high spatial resolution SERS imaging has enabled the monitoring of drug diffusion and detection of drug metabolites. Yang et al. used SERS for tracking the diffusion of multiple drugs, namely, 6-mercapotopurine (6MP) and methimazole (MMI), and monitoring their metabolisms in live cells.^52^ Fujita et al. used alkyne-tag SERS to develop a time-lapse 3D-imaging method for monitoring the uptake of drug molecules in live cells^53^ and analyzed the cellular uptake of drugs under various physicochemical conditions.^53^ Liz-Marzán et al.^45^ used a biocompatible gold nanorod-containing hydrogel-based scaffold for enhancing SERS signals to spatiotemporally detect and monitor drug uptake in 3D cancer cell models^45^ for application to cancer therapy by linking drug uptake with cell death.^45^ To monitor the cellular metabolism of a cancer therapeutic, Zhang et al.^54^ used label-free SERS and found that SERS spectral changes corresponded to changes in the adsorption of drug molecules on nanoparticle surfaces (Figure 3),^54^ revealing the cellular mechanisms of antitumor drugs.^54^ Other studies provide further insights into the application of vibrational spectroscopy, including SERS, for characterizing drug–cell interactions.^55^

### 3.3 Point-of-Care and Wearable SERS-Based Sensors for Biomarker, Metabolite, and Drug Detection and Monitoring

Emerging point-of-care and flexible wearable sensors show promising practical application prospects because they eliminate the requirement for centralized laboratory testing and enable the real-time monitoring of biomarkers, metabolites, and drugs *in situ*. One important consideration when developing a point-of-care sensor is choosing relevant analytes and health conditions that would benefit from frequent and quick monitoring for an adequate medical response. Examples of SERS-based point-of-care platforms, including wearable sensors, are discussed in this subsection.

**Figure 4:**
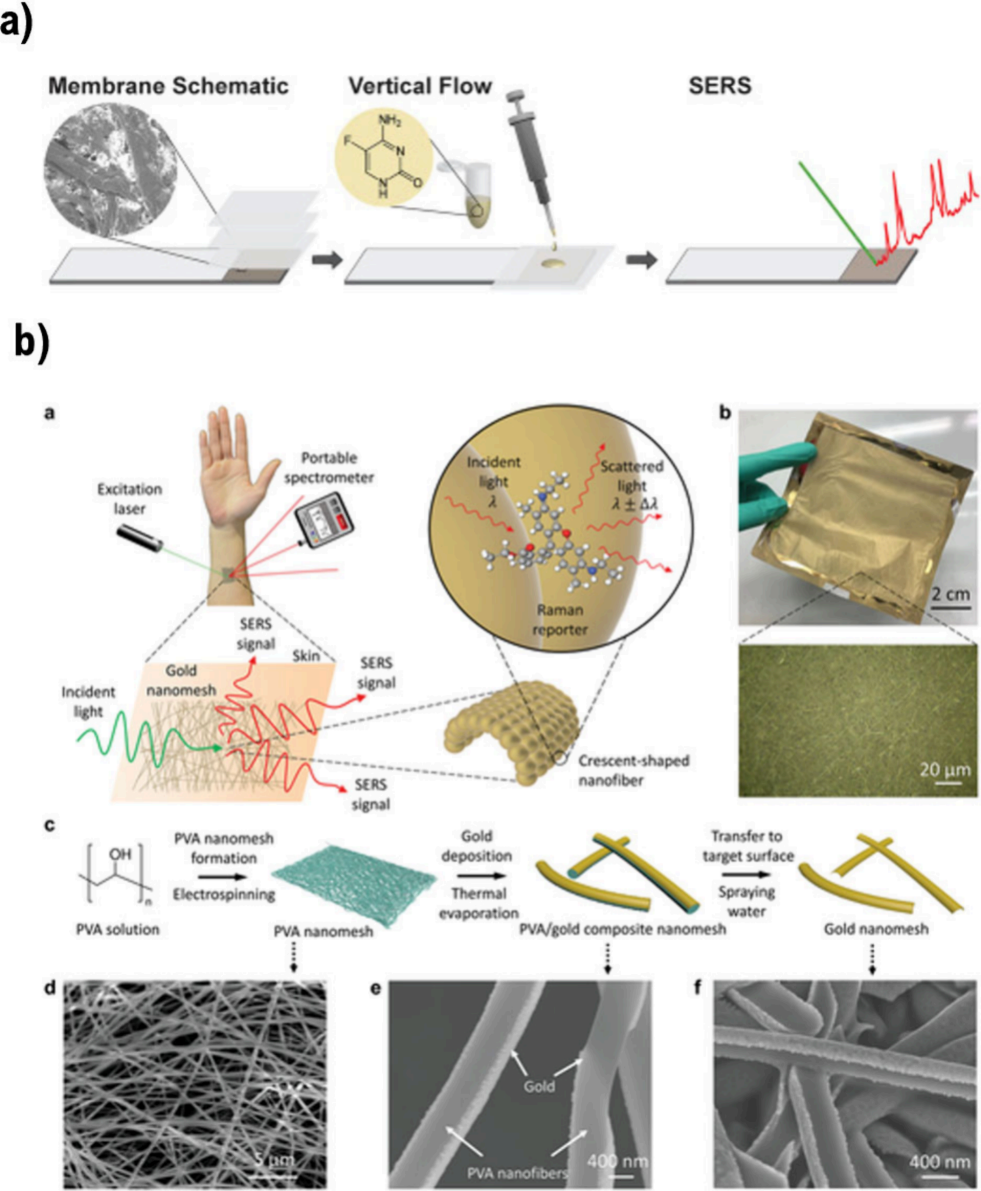
Wearable and point-of-care SERS-based sensors for detecting biomarkers, metabolites, and drugs. (a) Paper-based SERS substrate for point-of-care drug detection. Reproduced from ref (35). Copyright 2016, Elsevier B.V. (b) Concept of a SERS-based sensor, wearable directly on the skin, for detecting biomarkers and drugs. Reproduced from ref (59). Available under a CC-BY License. Copyright 2022, Limei Liu, Pablo Martinez Pancorbo, Ting-Hui Xiao, Saya Noguchi, Machiko Marumi, Hiroki Segawa, Siddhant Karhadkar, Julia Gala de Pablo, Kotaro Hiramatsu, Yasutaka Kitahama, Tamitake Itoh, Junle Qu, Kuniharu Takei, and Keisuke Goda. Advanced Optical Materials, Wiley-VCH GmbH.

Paper-based SERS substrates are among the most widely used point-of-care sensors for detecting and monitoring biomarkers, metabolites, and drugs.^56^ Torul et al. and Kim et al. demonstrated paper-based SERS platforms for quantifying glucose concentrations in blood samples and detecting prenatal disease biomarkers in human amniotic fluids, respectively.^56,57^ In addition, Berger et al. used a paper-based SERS substrate for detecting and monitoring therapeutic drugs, specifically flucytosine, a chemotherapeutic, in serum samples (Figure 4a).^35^ These paper-based point-of-care SERS sensors offer potential advancements in providing information required for personalized treatment or quick medical intervention using only drops of body fluids.

Flexible wearable SERS sensors have been developed to detect various disease-related biomarkers and metabolites associated with various health conditions, such as cancer, diabetes, and glaucoma.^58^ In addition, wearable SERS-based sensors offer opportunities for the continuous monitoring of relevant molecules. Compared with rigid sensors, flexible sensors are very advantageous because they can be attached to surfaces possessing flexible structures^58^ Liu et al. developed a scalable and wearable SERS sensor for detecting sweat biomarkers, such as ascorbic acid and urea, and drugs (Figure 4b).^59^ Similarly, Wang et al. designed a metamaterial-based wearable SERS sensor for monitoring drug concentrations and pH changes in sweat, providing insights into individual drug uptake and metabolic rates.^60^ Additionally, Jeong et al. devised a contact lens-based SERS sensor for monitoring glucose concentrations in tears.^61^

## 4 Conclusions

As personalized medicine and drug discovery advance, the integration of innovative technologies is essential for overcoming existing challenges. SERS is a promising technique, offering ultrasensitivity, rapid analysis, and compatibility with small amounts of liquid samples, among other advantages. However, the main challenges hindering the practical application of SERS are substrate irreproducibility and the variabilities that arise when working with complex biological samples. Masson contributed a valuable editorial note discussing challenges for benchmarking SERS.^62^ Despite its existing limitations, the examples covered in this Mini-Review showcase the potential of SERS to offer tailored solutions for overcoming the challenges faced in drug discovery and personalized medicine. The use of chemical modifications, separation methods, and statistical analysis or artificial intelligence algorithms is shown to offer a solution for near future real world applications of SERS despite its current reproducibility limitations. We expect SERS to play a significant role in the fields of personalized medicine and drug discovery.
